# Dairy intake screener as web‐based application is reliable and valid

**DOI:** 10.1002/fsn3.4187

**Published:** 2024-06-04

**Authors:** Monique C. Piderit, Zelda White, Piet J. Becker, Friedeburg A. M. Wenhold

**Affiliations:** ^1^ Department of Human Nutrition, Faculty of Health Sciences University of Pretoria Pretoria South Africa; ^2^ Research Office, Faculty of Health Sciences University of Pretoria Pretoria South Africa

**Keywords:** Dairy Diary, dairy intake screener, dietary screener, reliability, validity

## Abstract

The “Dairy Diary” is a user‐friendly web‐based dairy intake screener. The reliability and validity are unknown. We aimed to evaluate the screener in terms of test–retest reliability and comparative validity. In a diagnostic accuracy study, a purposefully recruited sample of 79 (age: 21.6 ± 3.8 years) undergraduate dietetics/nutrition students from three South African universities completed 3 non‐consecutive days of weighed food records (reference standard) within a seven‐day period (comparative validity), followed by two administrations, 2 weeks apart, of the screener (index test) (reliability). For the four dairy product serving scores (PSSs) and the summative dairy serving scores (DSSs) of the screener and the food records, t‐tests, correlations, Bland–Altman, Kappa, McNemar's, and diagnostic accuracy were determined. For reliability, mean PSSs and DSSs did not differ significantly (*p* > .05) between the screener administrations. The mean PSSs were strongly correlated: milk (*r* = .69; *p* < .001), maas (fermented milk) (*r* = .72; *p* < .001), yoghurt (*r* = .71; *p* < .001), cheese (*r* = .74; *p* < .001). For DSSs, Kappa was moderate (*k* = 0.45; *p* < .001). Non‐agreeing responses suggest symmetry (*p* = .334). For validity, the PSSs of the screener and food records were moderately correlated [milk (*r* = .30; *p* = .0129), yoghurt (*r* = .38; *p* < .001), cheese (*r* = .38; *p* < .001)], with *k* = 0.31 (*p* = .006) for DSS. Bland–Altman analyses showed acceptable agreement for DSSs (bias: −0.49; 95% CI: −0.7 to −0.3). Categorized DSSs had high sensitivity (81.4%) and positive predictive value (93.4%), yet low specificity (55.6%) and negative predictive value (27.8%). The area under the receiver operating characteristic curve (0.7) was acceptable. The “Dairy Diary” is test–retest reliable with moderate comparative validity to screen for dairy intake of nutrition‐literate consumers.

## Graphical abstract

The “Dairy Diary” is a user‐friendly web‐based dairy intake screener. The reliability and validity are unknown. We aimed to evaluate the screener in terms of test–retest reliability and comparative validity. The “Dairy Diary” is test–retest reliable and has moderate comparative validity to screen for dairy intake in groups.

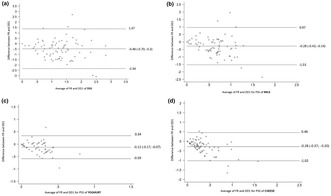

## 1 INTRODUCTION

Dietary assessment forms part of nutrition assessment, which includes the interpretation of dietary, laboratory, anthropometric, and clinical data to determine the nutritional status of individuals or populations (Field & Hand, 2015). Food frequency questionnaires (FFQs), 24‐h recalls, and food records are used for a comprehensive assessment of diet (Bailey, 2021).

When time and other resource constraints limit comprehensive dietary assessment, screening may be favored. Nutrition screening identifies an individual who is malnourished or is at risk of malnutrition to determine if further comprehensive nutrition assessment is required (Mueller et al., 2011). Despite overlaps, nutrition screening is separate from and different from nutrition assessment (Field & Hand, 2015; Swan et al., 2017) with the latter serving as a trigger in the nutrition care process for a more comprehensive assessment (Charney, 2008; Field & Hand, 2015; Swan et al., 2017). Dietary screening is typically achieved using short questionnaires and screeners (Charney, 2008). Such tools may take the basic form of a FFQ, adapted to be interpretable, for example, through a scoring system to identify the presence or absence of dietary risk. Dietary screeners should be cost‐effective, easy, and quick to use, with at least a high sensitivity for early detection of nutrition risk. However, in resource‐limited settings, high specificity (i.e., fewer false positives) may be favored in some instances (Trevethan, 2017). Regardless, it remains desirable for such tools to assess diet quality with reasonable accuracy in a short amount of time (Springfield et al., 2020).

Already a decade ago, individuals were within arm's reach of a mobile phone 50% of the time (Dey et al., 2011). This potentially drove the trend to access health‐ and nutrition‐related information via mobile applications (apps) (Chen et al., 2018; Krebs & Duncan, 2015). In South Africa, mobile app downloads are high (Nkume, 2018). The uptake of mobile technology highlights a significant opportunity to impact health behavior (Zhao et al., 2016), with technology‐based dietary screeners gaining favor over traditional (paper‐based) versions (Lucassen et al., 2021).

Despite consistent evidence of the positive role of dairy for health (Weaver, 2014), dairy is the most commonly deficient food group in South Africa (Mchiza et al., 2015). Internationally, dairy intake screeners have been developed and/or validated for populations in North America (Blalock et al., 2003; Gans et al., 2006; Gilsing et al., 2018; Hacker‐Thompson et al., 2009; Sebring et al., 2007), Australia (Clover et al., 2007; Gadowski et al., 2020; Hodge et al., 2000), Asia (Park et al., 2013; Tseng et al., 2021), the Netherlands (De Rijk et al., 2021; Gans et al., 2006; Goldbohm et al., 2011; Welten et al., 1995), and Poland (Martela et al., 2019), yet few of which are technology‐based (De Rijk et al., 2021; Gans et al., 2006; Hacker‐Thompson et al., 2009; Hodge et al., 2000). Neither a validated nor a technology‐based dairy intake screener is available in South Africa.

Thus, the aim of the Dairy Diary (Dairy Gives You Go, 2023) – as a general screener – is to presumptively identify the risk of low dairy intake at an individual level and a group level among South African adults so as to initiate timely intervention. The development has been described, and its usability has been established (Piderit et al., 2023). The reliability and validity of the dairy intake screener remain, however, unknown. We thus aimed to assess the agreement between the “Dairy Diary” (index test; screener) and 3‐day weighed food records (reference method) in dietetics/ nutrition students in South Africa to evaluate comparative validity (Gleason et al., 2010). Since reliability is a prerequisite for validity (Gleason et al., 2010), we included test–retest reliability assessment, which was defined as the reproducibility of the “Dairy Diary” scores when administered twice to the same participants.

## 2 MATERIALS AND METHODS

### 2.1 The “Dairy Diary”: Index test

The “Dairy Diary” is a self‐administered dietary screener with the structure of a quantitative FFQ, developed as a web‐based mobile app and accessible via an internet‐enabled smartphone, tablet, laptop, or computer (https://www.dairygivesyougo.co.za/dairy‐diary). The screener focuses on four commonly consumed dairy products in South Africa, each with two forms: milk (reduced fat or full cream), a local fermented milk, maas (reduced fat or full cream), yoghurt (plain or flavored), and cheese (hard or soft), resulting in an eight‐item food list. Reduced fat included fat‐free and low‐fat dairy products.

A product serving score (PSS) is calculated for each dairy product. The daily serving score (DSS) is the sum of the four PSSs. Guided by recommendations to consume at least two servings of dairy per day (Weaver, 2014), the DSS is classified into two categories: <2 servings daily or ≥2 servings daily.

### 2.2 Three‐day weighed food records: Reference standard

Food records were chosen as the reference standard, having an independent error structure compared to the FFQ format of the index test (Gleason et al., 2010). Using a digital scale and standardized template, participants completed 3 days of weighed food records (FR1, FR2, and FR3) on 2 non‐consecutive weekdays and 1 weekend day within a 7‐day period. Participants were provided with written and audio‐visual instruction and demonstration (MP4 video) on keeping a food record, including avoidance of changes in habitual diet, the immediate recording of all foods, beverages, and supplements consumed in a full 24‐h period, and nonedible parts to be weighed and indicated using the tare/zero function on the scale. For composite dishes, participants were asked to document and submit all ingredients and preparation methods.

### 2.3 Study design

The reporting of this diagnostic accuracy study to assess comparative validity was guided by the Standards for Reporting of Diagnostic Accuracy Studies (STARD) checklist (Cohen et al., 2016). The screener was also assessed in terms of test–retest reliability.

#### 2.3.1 Sample size, recruitment, and study population

The sample size was calculated using nQuery (version 8.3.0.0). For an assumed proportion of 60% of the population meeting dairy intake recommendations of ≥2 servings per day in the 3‐day weighed food records, a sample of at least 78 would have 90% power to reject the null hypothesis.

**FIGURE 1:**
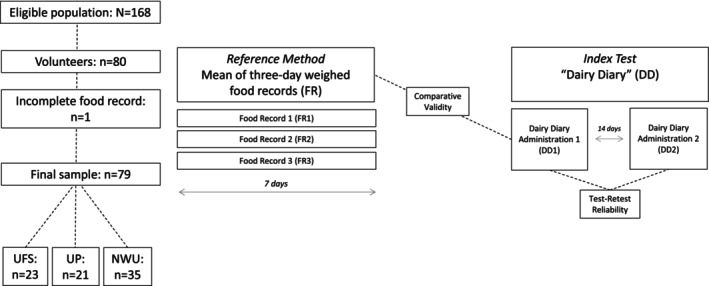
Flow diagram of study. DD, mean of the two administrations of the “Dairy Diary”; DD1, first administration of the “Dairy Diary”; DD2, second administration of the “Dairy Diary”; FR, mean of the 3‐days of food records; FR1, food record 1; FR2, food record 2; FR3, food record 3; NWU, North West University; UFS, University of the Free State; UP, University of Pretoria.

Recruitment took place between April 2020 and September 2021. Participants were conveniently recruited from an eligible population of 168 undergraduate dietetics/nutrition students from three universities in three provinces of South Africa (University of the Free State [UFS], University of Pretoria [UP], and North West University [NWU]). Participants were independently recruited by lecturers at each university in contact sessions (remotely due to COVID‐19, or in person). Inclusion criteria included access to a computer and/or smartphone and the internet. Data cleaning removed participants with incomplete 3‐day food records (*n* = 1). A final sample of 79 (47%) participants (first year: *n* = 11; second year: *n* = 40; third year: *n* = 28) was retained for analyses (Figure 1).

#### 2.3.2 Test–retest reliability

Test–retest reliability was evaluated by comparing the PSSs of each dairy product and the DSSs achieved in the first administration of the screener (DD1) to the corresponding scores in the second administration (DD2). According to Magarey et al. (2014), a time interval of 2 weeks was chosen between the two administrations (Figure 1). To minimize recall bias during the completion of the screener, data collection of the food records took place prior to the two administrations of the screener. Oral instruction (MP4 video) was provided to participants. Data were collected via Qualtrics (a secure, web‐based survey tool) interconnected to the online screener. Before the first administration, information on demographics (e.g., age, self‐reported weight and height, sex), perceived health status, and mobile app usage was also collected. To further reduce respondent memory bias, the final score of the screener (i.e., DSSs of DD1) was automatically blinded to participants to not influence the subsequent administration (Gleason et al., 2010).

#### 2.3.3 Comparative validity

Comparative validity was determined by comparing the DSSs and PSSs from the first administration of the screener (DD1) against the mean DSSs and corresponding PSSs of the 3‐day weighed food records. We used the first administration of the “Dairy Diary” to reduce recall bias from previous exposure to the dietary screener (Figure 1). The mean time interval between the completion of food records and the first administration of the screener was 13.1 days (Min–Max: 3–41 days). It was assumed that the usual intake of dairy was not seasonal.

#### 2.3.4 Ethical approval and informed consent

The study was approved by the UP Faculty of Health Sciences Research Ethics Committee (705/2018), NWU Health Research Ethics Committee (NWU‐00461‐19‐S1), and UFS Department of Human Nutrition and Dietetics. Informed consent at each data collection point, assurance of confidentiality, and blinding of recruiters (lecturers) to participation were included. Participants voluntarily provided contact details for individual feedback on their personal DSSs.

#### 2.3.5 Data management

For the dairy intake screener, data from Qualtrics were exported to Microsoft (MS) Excel format and cleaned for incomplete responses. The BMI (kg/m^2^) was calculated as self‐reported weight (kg) divided by self‐reported height squared (m^2^).

For comparison of the PSSs and DSSs of the food records and screener, the following was done. From the food records, raw data on the recorded portion size of dairy products consumed (milk: mL; maas, yoghurt, and cheese: g) were captured in MS Excel and added per day. These quantities were converted into daily serving equivalents using a reference serving of 250 mL for milk, 250 mL for maas, 200 mL for yoghurt, 30 g for hard cheese (e.g., cheddar, gouda, mozzarella), and 60 mL for soft cheese (e.g., cottage cheese, ricotta cheese), i.e., amounts containing 300 mg of calcium (U.S. Department of Agriculture and U.S. Department of Health and Human Services, 2020). The PSSs for each dairy product were summed to calculate the food record PSSs and DSSs. This was repeated for each of the three food records. The mean of the PSSs and DSSs of the three food records (FR) were calculated, and DSSs were also categorized as <2 servings daily or ≥2 servings daily. Dairy products contribute 60% (van Rossum et al., 2020) to 75% (Cormick & Belizan, 2019) of dietary calcium intake. Considering non‐dairy food sources of calcium as contributors to meeting calcium requirements, we categorized dairy intake of ≥2 servings per day as adequate for this study.

For quality control, data from food records were captured in MS Excel by the researcher (MP) and an independent research assistant, both registered dietitians with post‐graduate qualifications applying pre‐set coding rules. This was followed by the automated conversion of dairy product volumes to PSSs and DSSs. Cross‐checking of data included conditional formatting in MS Excel to automatically alert for data capturing differences, verified by the researcher (MP).

#### 2.3.6 Statistical analysis

Statistical analyses were performed with Stata (Release 17.0, College Station, Texas; StataCorp LLC, 2021). A *p*‐value of <.05 was considered statistically significant. Background characteristics were described. For reliability and validity assessment, multiple statistical analyses were performed (Lombard et al., 2015), including mean differences, paired t‐tests, and Pearson rank correlations for continuous data, and Kappa statistics for categorical data. For test–retest reliability, McNemar's test for symmetry was additionally performed on categorized DSSs. For validity assessment, agreement between the DSSs of DD1 and the mean DSSs of the three food records (FR) was verified with Bland–Altman plots. Sensitivity, specificity, predictive values, odds ratios, and Receiver Operating Characteristics (ROC) were used to quantify the diagnostic ability of the categorized DSSs of the “Dairy Diary.”

Correlation strength was described as poor (*r* < .2), moderate (*r* = .2–.6), and strong (*r* > .6) (McNaughton et al., 2007; Schumacher et al., 2016). The strength of agreement for Kappa was described as poor (*k* < 0), slight (*k* = 0.01–0.2), fair (*k* = 0.21–0.40), moderate (*k* = 0.41–0.60), strong (*k* = 0.61–0.80), and almost perfect (*k* = 0.81–1.0) (Landis & Koch, 1977). For Bland–Altman analyses, a clinically relevant a priori acceptable level of error (Hanneman, 2008) was defined as 0.5 dairy servings (i.e., 75% of the recommended dairy intake of ≥2 servings per day). For ROC, the area under the curve was 1.0 for a perfect test and 0.5 for a poor outcome (Soreide, 2009).

## 3 RESULTS

### 3.1 Description of participants

From a total of 80 volunteers, 79 (98.8%) participants completed 3‐day weighed food records and two administrations of the screener (Figure 1). Participants had a mean ± SD age of 21.6 ± 3.8 years and a BMI of 22.7 ± 3.1 kg/m^2^. Most participants (98.7%) were female, and 78.5% had a healthy (18.5–24.9 kg/m^2^) BMI (World Health Organization, 2004). Most (62.0%) completed the screener on a smartphone, and almost two‐thirds (58.2%) reported being “very healthy” (Table 1).

**TABLE 1:** Demographic information of study participants (*N* = 79).

Background characteristic	*n*	%
Sex		
Female	78	98.7
How did you complete the “Dairy Diary”?		
On a desktop/laptop	29	36.7
On a smartphone	49	62.0
On a tablet	1	1.3
In general, how is your health?		
Very healthy	46	58.2
Somewhat healthy	32	40.5
Not healthy	1	1.3

### 3.2 Test–retest reliability

When comparing DD1 and DD2, there were no significant differences between all the corresponding PSSs [milk (*p* = .663), maas (*p* = .342), yoghurt (*p* = .866), cheese (*p* = .823)], as well as DSSs (*p* = .679) (Table 2). For all four dairy products, the correlation coefficients between the first and second administrations of the PSSs were strong and statistically significant (*r* > .6; *p* < .001). The Kappa coefficient indicated moderate agreement between the categorized DSSs (*p* < .001). In relation to the categorized DSS, the McNemar test showed symmetry (*p* = .334).

**TABLE 2:** Test–retest reliability of components of the “Dairy Diary” (*N* = 79).

“Dairy diary” component	Scores			Reliability indicators		
	DD1 mean ± SD	DD2 mean ± SD	Mean difference	*p*‐Value^a^	r	*p*‐Value^d^
PSSs						
Milk	0.75 ± 0.55	0.77 ± 0.49	−0.22	.663	.69^b^	<.001
Maas	0.01 ± 0.02	0.03 ± 0.21	−0.22	.342	.72^b^	<.001
Yoghurt	0.25 ± 022	0.25 ± 0.22	0.00	.866	.71^b^	<.001
Cheese	0.49 ± 0.43	0.50 ± 0.44	−0.01	.823	.74^b^	<.001
DSS, continuous	1.50 ± 0.82	1.53 ± 0.87	0.02	.675	.68^b^	<.001
DSS, categorized					.45^c^	<.001

Abbreviations: DD1, first administration of the “Dairy Diary”; DD2, second administration of the “Dairy Diary”; Mean difference: DD1 − DD2.

^a^ Paired *t*‐test.

^b^ Pearson.

^c^ Kappa.

^d^ Level of significance for *r*.

### 3.3 Comparative validity

When comparing DD1 and FR, there were significant differences (*p* < .05) in mean intakes for all dairy products and the DSSs (Table 3). Pearson correlation coefficients were significant (*p* < .05 for all) and moderate for milk (*r* = .30), yoghurt (*r* = .38), and cheese (*r* = .38). The Kappa coefficient was fair for DSS (*k* = 0.31).

**TABLE 3:** Product serving score (PSS) and daily serving score (DSS) of the “Dairy Diary” compared to the food records (FR) (*N* = 79).

Components of dairy intake	Scores					Validity indicators		
	“Dairy diary”	Food records				*p*‐Value^c^	r	*p*‐Value^f^
	DD1	FR1	FR2	FR3	FR			
	Mean ± SD	Mean ± SD	Mean ± SD	Mean ± SD	Mean ± SD			
Milk								
PSS^a^	0.77 ± 0.60	—	—	—	0.48 ± 0.40	<.001	.30^d^	.0129
Maas								
PSS^a^	0.00 ± 0.00	—	—	—	0.00 ± 0.00	<.001	No estimate possible: lack of variation	
Yoghurt								
PSS^a^	0.22 ± 0.41	—	—	—	0.13 ± 0.16	<.001	.38^d^	.0005
Cheese								
PSS^a^	0.42 ± 0.60	—	—	—	0.20 ± 0.23	<.001	.38^d^	.0007
DSS^b^, continuous	1.51 ± 0.88	1.02 ± 0.88	1.05 ± 0.88	0.97 ± 1.03	1.01 ± 0.71	<.001	.30^d^	.0073
DSS^b^, categorized	—	—	—	—	—	—	.31^e^	.0057

Abbreviations: DD1, first administration of the “Dairy Diary”; FR, Mean PSSs and DSSs for three food records: FR1 + FR2 + FR3/3.

^a^ Product of serving score and frequency score. Serving score: For each dairy product, the frequency (number of times) of consumption was assessed in four frequency categories: never, per day (0–3 times), per week (1–6 times), or per month (1–3 times). Each frequency category was converted into a daily intake. Frequency score: Scored daily intake based on 300 mg calcium equivalents (i.e., 250 mL for milk, 250 mL for maas, 200 mL for yoghurt, 40 g for hard cheese, and 60 mL for soft cheese).

^b^ Sum of the four product serving scores.

^c^ Paired *t*‐test comparing PSSs/DSSs to FR.

^d^ Pearson (continuous scores).

^e^ Kappa (categorized scores).

^f^ Level of significance for *r*.

**FIGURE 2:**
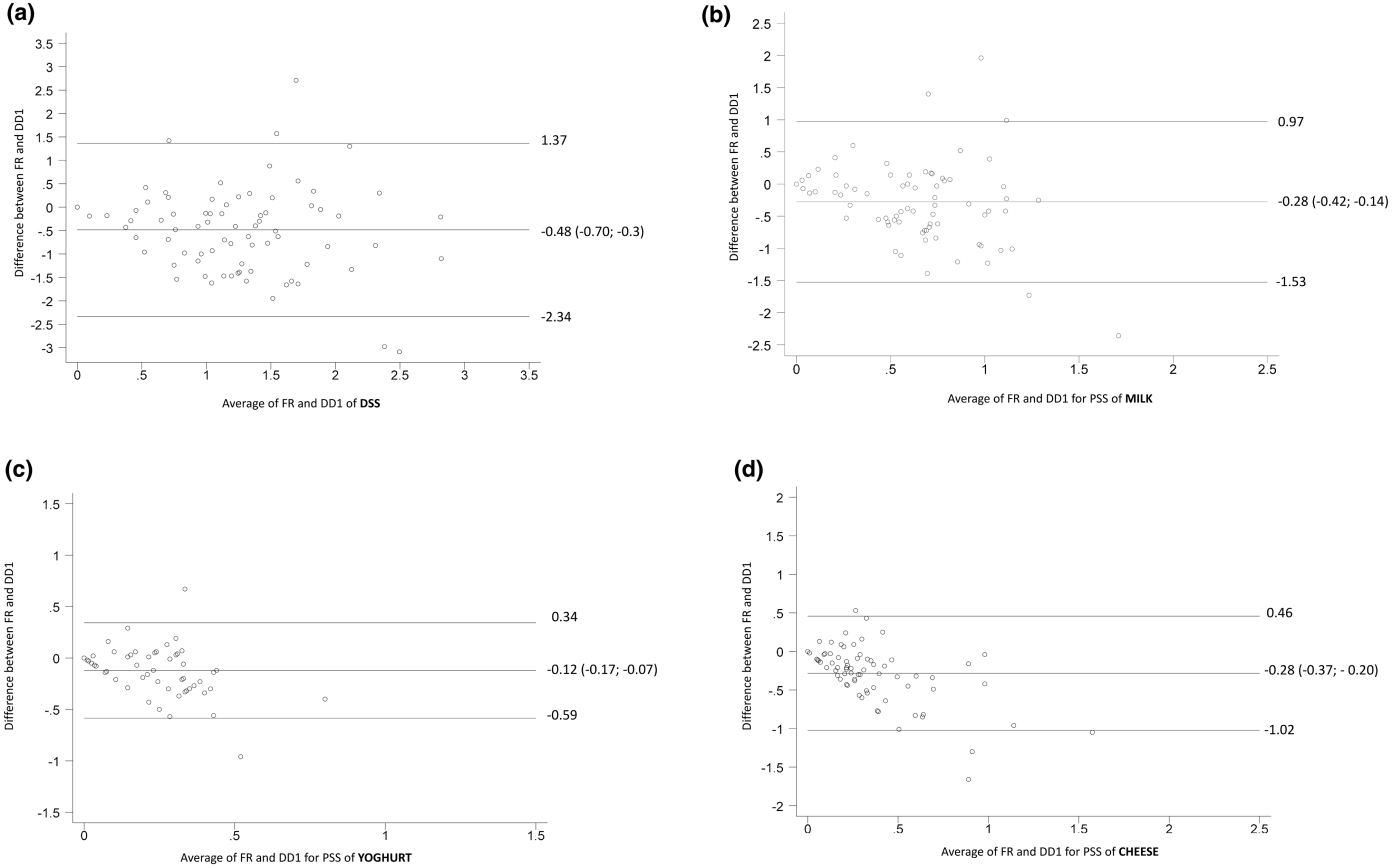
Bland–Altman plots for dairy serving score (DSS) and product serving score (PSS) of milk, yoghurt, and cheese (*n* = 79) including mean difference (bias) and limits of agreement (LOA; ±1.96 SD; 95% CI of the mean difference).

Agreement between the first administration of the screener and food records was assessed by Bland–Altman analyses. Figure 2 shows plots for PSSs milk, yoghurt, and cheese. No plot could be presented for maas due to a lack of variation in intake. For DSS, Bland–Altman analyses showed acceptable agreement (bias: −0.48; 95% CI: −0.7 to −0.3), yet considerable imprecision.

The parameters of diagnostic accuracy of the DSS of the screener relative to the DSS of the food records are shown in Table 4. Sensitivity (81.4%) and positive predictive value (PPV) (93.4%) were higher than specificity (55.6%) and negative predictive value (NPV) (27.8%), respectively. The area under the ROC curve was 0.7.

**TABLE 4:** Diagnostic accuracy of the categorized dairy serving score (DSS) of the “Dairy Diary” relative to the DSS of the weighed food records (*N* = 79).

Parameter of diagnostic accuracy	Value (95% CI)
Sensitivity	81.4% (70.3; 89.7)
Specificity	55.6% (21.2; 86.3)
Receiver operator characteristics (ROC): area under the curve	0.7 (0.51; 0.86)
Positive likelihood ratio (+)	1.83 (0.88; 3.84)
Negative likelihood ratio (−)	0.33 (0.16; 0.72)
Odds ratio (OR)	5.5 (4.4; 21.7)
Positive predictive value (PPV)	93.4% (84.1; 98.2)
Negative predictive value (NPV)	27.8% (9.7; 53.5)

## 4 DISCUSSION

The aim of the “Dairy Diary” as a general dairy intake screener is to classify individuals into those with and without low dairy intakes. For reliability assessment, mean PSSs and DSSs did not differ significantly between the two screener administrations. Supporting this, correlations were strong for milk, maas, yoghurt, and cheese. Similar correlations have been shown elsewhere for milk and cheese (Goldbohm et al., 2011; Welten et al., 1995). McNemar's test for symmetry showed no bias for the DSSs between the two administrations of the “Dairy Diary,” suggesting that the proportion of individuals who underestimated dairy intake was comparable to the proportion who overestimated their intake in the two administrations. Multiple statistical analyses thus concur with and support test–retest reliability.

For validity assessment, the PSSs of the screener and food records were moderately correlated for milk, yoghurt, and cheese, with fair agreement for the categorized DSS. Based on a priori limits of agreement of 0.5 servings, the Bland–Altman plot for DSS showed, on a group level, acceptable accuracy between DSS for the “Dairy Diary” and food records, making the “Dairy Diary” appropriate for research studies where group means are important.

We further quantified the diagnostic ability of the “Dairy Diary.” Sensitivity referred to the ability of the “Dairy Diary” to correctly identify participants consuming <2 servings of dairy per day. Specificity referred to the ability of the “Dairy Diary” to correctly identify participants consuming ≥2 servings of dairy per day. Our results show high sensitivity and low specificity, aligned to sensitivity and specificity values reported for other calcium‐ and food‐based screeners that include dairy products. In such studies, sensitivity values ranged from 56% (Tseng et al., 2021) to 97% (Martela et al., 2019) and specificity values from 12% (Martela et al., 2019) to 87% (Montomoli et al., 2002).

The high sensitivity of the “Dairy Diary” suggests the screener can correctly identify participants not meeting dairy intake recommendations, at the expense of low specificity, where the screener is less likely to correctly identify those meeting dairy intake recommendations. It is argued that high sensitivity and high specificity are not feasible (Charney, 2008; Field & Hand, 2015), with a pattern of higher sensitivity and lower specificity (and vice versa) to be expected (Gleason et al., 2010). A balance must be struck, and we reason that the need to correctly identify low dairy intakes (sensitivity) takes precedence over misclassifying those who consume sufficient dairy (specificity) to trigger entry into the nutrition care process for comprehensive dietary assessment (Swan et al., 2017). It would be undesirable to have a high rate of false negatives (i.e. failure to identify those who are at risk of low dairy intakes), given well‐established evidence that dairy plays a positive role in managing non‐communicable diseases (Aljuraiban et al., 2019; Bhupathi et al., 2020; Guo et al., 2019; Thorning et al., 2017), and helping to meet gap nutrient intakes as a surrogate marker of diets higher in nutritional quality (Weaver, 2014).

Last, we supplemented the diagnostic ability of the “Dairy Diary” with predictive values, acknowledging that such values are related to population prevalence (Gleason et al., 2010) and the possibility that dietetics/nutrition may not be perfectly reflective of the general higher income population of South Africa. Nonetheless, the large proportion of participants with a daily dairy intake below two servings a day limits this threat. Given that the screener is also intended to create awareness of low dairy intakes, we likewise favored higher PPV. PPVs and NPVs are suggested as statistical tests when screening is likely to be conducted by a non‐nutrition professional (Field & Hand, 2015). High PPV and low NPV of the Dairy Diary was reported in this research. Having included these statistical tests affirms that the Dairy Diary study may be conducted by other trained professionals. Nonetheless, it is argued that predictive values need not always be high (Trevethan, 2017), as predictive values are dependent on the population being tested and related to disease prevalence (Gleason et al., 2010). In this research, consistent with population‐based data on dairy intake in South Africa (Mchiza et al., 2015), dairy intakes lower than the recommended ≥2 servings per day for both the Dairy Diary and weighed food records were reported. The observed pattern of a higher PPV than NPV implies that false positives are minimized, which is desirable when the risk of poor dairy intake is not identified and entry into the nutrition care process is delayed. In the context of the positive role that dairy plays in health, a dairy intake screener that delays the identification of low dairy intake is more of a concern than a screener that overidentifies high dairy intake. These predictive values would, however, change should the Dairy Diary be validated in a different population group, such as one with a higher dairy intake.

Furthermore, on a group level, the Bland–Altman plot for DSS showed acceptable accuracy and limited bias between DSS for the Dairy Diary and food records. Taken together, this supports that the Dairy Diary is appropriate for use as a dairy intake screener in research studies where group values are important.

In assessing the high positive likelihood ratio (>1) and low negative likelihood ratios (<1), results suggest that the “Dairy Diary” is effective at establishing low dairy intakes whilst also being effective at ruling out low dairy intakes (i.e., ≤2 servings of dairy per day). Furthermore, an OR of 5.5 suggests that the odds of low dairy intake in those consuming <2 servings of dairy per day are greater than the odds of low dairy intake in those who consume ≥2 servings of dairy per day. The area under the ROC of 0.7 suggested that the “Dairy Diary” had a moderate predictive ability. Previously, ROC analyses have been done on a 6‐item calcium‐intake screener (Tseng et al., 2021), yet the area under the curve was not reported.

In general, our results show that the first administration of the “Dairy Diary” tended to have higher DSSs (and PSSs) compared to food records. Since the “Dairy Diary” reflects *usual* dairy intake, whereas weighed food records capture *actual* dairy intake within a 7‐day period, perfect agreement may be considered unrealistic. It is, however, also conceivable that the expert‐predefined serving sizes in the “Dairy Diary” may partly explain the overestimated portion sizes in the screener. Improvements in the performance of FFQs when population‐relevant usual portion sizes are included, have been reported (Illner et al., 2012; Molag et al., 2007), pointing to the need for locally verified actual dairy portion sizes in the screener.

Strengths of this study include self‐administration of the screener and food records (minimizing social desirability bias), the 2‐week time interval between the two administrations of the screener (minimizing memory and recall bias), and participant blindness to the outcome of the screening (minimizing influence on the second administration). In the absence of a feasible gold standard, 3‐day weighed food records, consistent with other validity studies (Clover et al., 2007; Gans et al., 2006; Goldbohm et al., 2011; Hacker‐Thompson et al., 2009; Martela et al., 2019; Sebring et al., 2007), were used. Food records have an inherently different error structure compared to the FFQ format of the “Dairy Diary,” minimizing systematic error (Gleason et al., 2010). We addressed random error (linked to day‐to‐day variation) with repeated (three) and non‐consecutive (2 weekdays and 1 weekend day) weighed food records to mimic usual intake, assuming dairy intake was not seasonal. Systematic error was managed with standardized instructions for participants on how to record food intake. We also elected not to exclude non‐dairy‐consuming participants as outliers, which may have led to inflated estimates of the reliability and validity of the “Dairy Diary,” weakening the diagnostic accuracy of this study.

In terms of the screener, recommendations include the use of the ROC analysis to optimize cut‐off values to improve sensitivity and specificity values. In our study, we did not attempt this, as this may differ depending on the prevalence rates of low dairy intake within the population. The “Dairy Diary” was developed for high‐income South African adults, yet the inclusion of maas, a traditional fermented milk, may have been less relevant to the young sample population (university students) included in our study. Reconsidering the role of maas in the screener may be necessary, or, alternatively, we recommend redefining the target market.

We acknowledge that the assumption that dietetics/nutrition students at universities are representative adults of higher income in South Africa could be challenged. Furthermore, we acknowledge that volunteer participants in dietetics/nutrition may naturally be more food‐aware and healthier than the general population (Clover et al., 2007), leading to a potential selection bias that could limit the generalizability of this study. While the assumption of 60% of the population meeting dairy intake recommendations was not met, our sample of 79 remained aligned to the recommended 50–100 participants in validation studies (Cade et al., 2004). The assessment of the validity of a dietary screening tool is ongoing, and further studies exploring the applicability of the “Dairy Diary” in other population groups (including males, participants without a nutrition background, younger children, and older adults) will be valuable.

The practical implications of utilizing mobile apps for public health initiatives must be mentioned. This may include enhanced accessibility to health information, improved user engagement, and the potential for real‐time data collection and analysis. A systematic review on mobile app‐based health promotion reported better health outcomes for mobile users compared to non‐user (Lee et al., 2018).

In nutrition research, the assessment of usual or true dietary intake will always be a challenging yet necessary undertaking, driving continued discussion and debate on the most accurate method for assessing dietary intake (Bingham, 2002). Since no gold standard exists, a measure of validity can only be comparative and assessed by another method deemed to be superior (Ortega et al., 2015). Three‐day (non‐consecutive) weighed food records were used as a reference standard in the validity sub‐study – a dietary assessment method commonly used in validation studies (Ortega et al., 2015). Food records have a great degree of demonstrated validity, even if they are not an exact measure of usual dietary intake (Gleason et al., 2010). To address challenges, the use of other reference standards, independent of random and systematic errors, should be considered. Such limitations can be overcome with the use of biomarkers as a reference standard to objectively assess food consumption with independence and without the bias of (subjective) self‐reported dietary intake (Bingham, 2002; Pico et al., 2019). That said, the use of biomarkers as a reference standard would have been limited as, to our knowledge, there are no biomarkers for dairy as a food group. Rather, biomarkers for dairy intake are limited to assessing dairy fat using certain short‐chain fatty acids and amino acids (Bertram et al., 2007; Brevik et al., 2005; Pedersen et al., 2011; Riserus & Marklund, 2017; Zheng et al., 2015). However, the use of a biomarker in this study would have been challenged by budget and logistics related to the large geographical distance between participants in the validity sub‐study across three South African provinces. For these reasons, biological specimens were not considered to serve as biomarkers or the reference standard for this study.

A dairy intake screener that is user‐friendly and valid may help support and promote current low dairy intakes in South Africa by alerting the consumer to poor intakes, thereby providing a platform to emphasize dairy‐based nutrition education. Further research could address validating the Dairy Diary in other groups, such as young children, the elderly, and lower income groups, which may help create dairy intake awareness across larger segments of South Africa.

## 5 CONCLUSION

While individual‐level error must be expected, the Dairy Diary has the potential to be comparatively valid to screen for dairy intake in groups, as in research studies. The high sensitivity suggests that the screener can correctly identify participants not meeting dairy intake recommendations.

## AUTHOR CONTRIBUTIONS

**Monique C. Piderit:** Formal analysis (equal); funding acquisition (equal); investigation (equal); methodology (equal); validation (equal); visualization (equal); writing – original draft (equal); writing – review and editing (equal). **Zelda White:** Supervision (supporting); writing – review and editing (supporting). **Piet J. Becker:** Formal analysis (lead). **Friedeburg A. M. Wenhold:** Supervision (lead); writing – review and editing (lead).

## CONFLICT OF INTEREST STATEMENT

FW and ZW are members of the Technical Advisory Committee of the CEP of Milk SA. The working group that developed the “Dairy Diary” was blinded to the algorithm used to calculate the DSS.

## Data Availability

The dataset used and analyzed during the current study is available from the University of Pretoria on reasonable request.
